# Chaotic Homes and Children’s Disruptive Behavior: A Longitudinal Cross-Lagged Twin Study

**DOI:** 10.1177/0956797611431693

**Published:** 2012-06

**Authors:** Sara R. Jaffee, Ken B. Hanscombe, Claire M. A. Haworth, Oliver S. P. Davis, Robert Plomin

**Affiliations:** 1 Institute of Psychiatry, Medical Research Council Social, Genetic & Developmental Psychiatry Centre, King’s College London; 2 The Wellcome Trust Centre for Human Genetics, University of Oxford; 3 European Bioinformatics Institute, Wellcome Trust Genome Campus, Hinxton, Cambridge, England

**Keywords:** household chaos, home environment, disruptive behavior, conduct problems, hyperactivity, gene-environment correlation, behavioral genetics, childhood development

## Abstract

Chaotic home lives are correlated with behavior problems in children. In the study reported here, we tested whether there was a cross-lagged relation between children’s experience of chaos and their disruptive behaviors (conduct problems and hyperactivity-inattention). Using genetically informative models, we then tested for the first time whether the influence of household chaos on disruptive behavior was environmentally mediated and whether genetic influences on children’s disruptive behaviors accounted for the heritability of household chaos. We measured children’s perceptions of household chaos and parents’ ratings of children’s disruptive behavior at ages 9 and 12 in a sample of 6,286 twin pairs from the Twins Early Development Study (TEDS). There was a phenotypic cross-lagged relation between children’s experiences of household chaos and their disruptive behavior. In genetically informative models, we found that the effect of household chaos on subsequent disruptive behavior was environmentally mediated. However, genetic influences on disruptive behavior did not explain why household chaos was heritable.

Although children’s social environment—comprising day-to-day interactions between children and caregivers—has long been a focus of research on children’s development, researchers have only recently begun to study children’s physical environment (G. W. Evans, 2006; G. W. Evans, Gonnella, Marcynyszyn, Gentile, & Salpekar, 2005). This research has shown that children raised in chaotic homes—characterized by noise, overcrowding, and a lack of order—tend to score lower on tests of cognitive ability and self-regulatory capabilities, have poorer language abilities, and score higher on measures of problem behaviors and learned helplessness than do children raised in less chaotic environments (G. W. Evans et al., 2005; Hanscombe, Haworth, Davis, Jaffee, & Plomin, 2011). These associations have been demonstrated prospectively and controlling for familywide characteristics (e.g., income, maternal depression) that could potentially confound the association (Deater-Deckard et al., 2009; Dumas et al., 2005).

Researchers have identified a number of mechanisms by which being raised in chaotic homes could lead to relatively poor cognitive and behavioral outcomes in children. Parents in more crowded homes are less verbally responsive with their children than parents in less crowded homes are, and this accounts for the relatively low complexity of their own speech and, plausibly, their children’s speech (G. W. Evans, Maxwell, & Hart, 1999). Children—like adults—may respond to household chaos by learning to filter out unwanted stimuli and may then generalize this strategy to other settings (e.g., the classroom) in which it is less adaptive.

Although household chaos may be a cause of children’s poor developmental outcomes, it may also be a result of children’s behavior. On the face of things, this seems counterintuitive. Household chaos was originally conceptualized as a measure of the physical environment, comprising background noise, crowding, and foot traffic in the home (Wohlwill & Heft, 1987). However, features of the physical and social environment fall along a continuum, with one extreme reflecting inanimate, nonresponsive, background sources of stimulation (which are unlikely to be influenced by the child; e.g., traffic noise) and the other reflecting responsive, animate, and focal sources of stimulation, such as parental speech (which is more likely to be influenced by the child; Wachs, 1989). Some features of the environment will therefore combine characteristically physical and social elements. For example, the television could be at high volume because a child has ignored repeated requests to turn it down. The decibel level in a home could be high because children have not heeded requests to take noisy play outdoors.

The Confusion, Hubbub, and Order Scale (CHAOS; Matheny, Wachs, Ludwig, & Phillips, 1995) is a widely used measure of household chaos that combines physical and social elements. Items include, “It’s a real zoo in our home” and “You can’t hear yourself think in our home”—conditions that could be generated by a child’s behavior. Indeed, the CHAOS measure captures a broad construct of chaotic living conditions, characterized not only by factors such as noise and crowding, but also by qualities such as a lack of structure and routine (G. W. Evans et al., 2005). The fact that the CHAOS measure potentially captures effects of children on their environment raises questions about whether household chaos is a cause of children’s disruptive behavior or whether disruptive children create or perceive chaotic environments.

Recent quantitative genetics research has shown that although environmental factors largely explain why some children are more likely than others to perceive their homes as chaotic, genetic factors account for a significant 22% of the variation in these perceptions (Hanscombe, Haworth, Davis, Jaffee, & Plomin, 2010). But can these factors be identified? This would entail demonstrating that (a) some characteristic of the child predicts household chaos, (b) that characteristic is genetically influenced, and (c) genetic influences on that characteristic also account for genetic variation in household chaos.

We hypothesized that children’s disruptive behavior problems (e.g., poor conduct and hyperactivity-inattention), which are genetically influenced traits, would partially account for the heritability of household chaos. Given moderately strong correlations between social disadvantage and disruptive behavior problems (Duncan & Brooks-Gunn, 1997), disruptive children may experience their environments as being noisy, crowded, and lacking in structure. Additionally, it is possible that children’s disruptive behavior partly creates an environment that is noisy, in which it is difficult to concentrate, and in which children refuse to adhere to rules or routines related to television viewing, bedtimes, or mealtimes.

Results from other studies have identified parent- and child-driven effects in the relation between children’s disruptive behaviors and aspects of the family environment, such as parent-child conflict (Burt, McGue, Krueger, & Iacono, 2005) and parental negativity (Larsson, Viding, Rijsdijk, & Plomin, 2008). Thus, we also hypothesized that household chaos would have an environmentally mediated effect on children’s disruptive behavior.

## Method

### Sample

The sample was drawn from the Twins Early Development Study (TEDS; Oliver & Plomin, 2007). TEDS is a population-based longitudinal study of over 10,000 pairs of twins born in England and Wales from 1994 to 1996. Informed consent was obtained from the twins’ parents at each wave of assessment. The present study includes data from the TEDS assessments of twins at ages 9 and 12. The 1994 and 1995 birth cohorts were tested at age 9; all three birth cohorts were tested at age 12. In this study, the sample comprised 6,286 pairs—2,255 monozygotic (MZ) pairs, 2,051 dizygotic (DZ) same-sex pairs, and 1,980 DZ opposite-sex pairs—for whom data were available on at least one measure from at least one twin in a pair. All available data were used in the genetic analyses, which were performed using full-information maximum-likelihood estimation.

The TEDS sample used in our study is representative of the general population of children ages 9 and 12 in the United Kingdom. For example, United Kingdom census data for families with children indicate that 93% of children are White, 32% of mothers have at least one A level (Advanced Level General Certificate of Education exams usually taken at age 18), and 49% of mothers and 89% of fathers are employed. For the entire TEDS sample, 92% are White, 35% have mothers with A levels, and 43% of mothers and 92% of fathers are employed. For the TEDS sample who participated at age 9, 94% are White, 41% have mothers with A levels, and 46% of mothers and 93% of fathers are employed; for the TEDS sample who participated at age 12, 93% are White, 41% have mothers with A levels, and 47% of mothers and 93% of fathers are employed. Zygosity was assigned to the twins using a parent-rated instrument that yielded 95% accuracy when compared with zygosity established from DNA markers (Price et al., 2000); if there was any uncertainty, we conducted a follow-up assessment with DNA marker testing.

### Measures

#### CHAOS: Confusion, Hubbub, and Order Scale

At ages 9 and 12, children’s perceptions of household chaos were assessed with a short version of the CHAOS questionnaire (Matheny et al., 1995). The short form of CHAOS assesses the level of routine, noise, and general environmental confusion with six items (e.g., “I have a regular bedtime routine” [reverse-scored], “You can’t hear yourself think in our home”). The children rated whether each item was “not true,” “quite true,” or “very true.” At age 9 (α = .58) and age 12 (α = .57), responses to the items were averaged, with higher scores indicating more household chaos. CHAOS scores at both ages were normally distributed. The correlation between child-reported CHAOS scores at ages 9 and 12 was .43. Although child-specific measures were necessary to perform the present analyses, correlations between familywide parent reports and individual-specific child reports support the validity of child-reported CHAOS scores (α = .53 and α = .55 at 9 and 12 years, respectively).

#### Disruptive behavior

Conduct problems and hyperactivity-inattention were reported by parents when the twins were 9- and 12-years-old using the Strengths and Difficulties Questionnaire (SDQ; Goodman, 1997). The SDQ is a brief screening measure of children’s problem behaviors. Parents reported whether each of five items measuring conduct problems (e.g., “Generally obedient or usually does what adults request”) and five items measuring hyperactivity-inattention (e.g., “Easily distracted or concentration wanders”) were “not true,” “somewhat true,” or “definitely true” of their child. The SDQ subscales showed acceptable levels of internal consistency at both age 9 (conduct: α = .57; hyperactivity: α = .76) and age 12 (conduct: α = .55; hyperactivity: α = .76). The moderate internal consistency for conduct problems does not seem to be specific to our sample: A recent article exploring the validity and reliability of the SDQ scale in a Dutch sample (*N* = 562, mean age = 12.3 years) found the same internal consistency (α = .55) for the Conduct Problems subscale (Muris, Meesters, & van den Berg, 2003).

### Analyses

#### Phenotypic analyses

To test the cross-lagged effect of CHAOS scores at age 9 on disruptive behavior (conduct problems or hyperactivity-inattention) at age 12, we performed an ordinary least squares regression of the form,

$$Y_{\text{age}\;\text{12}}=b_{0}+b_{1}Y_{\text{age}\;\text{9}}+b_{2}X_{\text{age}\;\text{9}}+\varepsilon ,$$

where *Y* represents disruptive behavior and *X* represents CHAOS scores. To test for the reverse process, we reversed the variable order: CHAOS scores at age 12 were regressed on CHAOS scores at age 9 and disruptive behavior at age 9. The coefficient *b*_2_ measures the cross-lagged relation between disruptive behavior and CHAOS scores; *b*_1_ measures the effect within trait across time; *b*_0_ is the intercept.

#### Classical twin design

Comparison of MZ and DZ twins provides a method for estimating the genetic and environmental contributions to variance within and covariance between traits (Plomin, DeFries, McClearn, & McGuffin, 2008; Rijsdijk & Sham, 2002). MZ twins are 100% identical. DZ twins are 50% identical, on average, for the DNA that varies in humans. Thus, the extent to which MZ twins are more alike than DZ twins on any particular trait is due to their greater genetic relatedness. The twin model partitions the variance of a trait, or the covariance between traits, into additive genetic (*A*), shared (common) environmental (*C*), and nonshared environmental (*E*) components (D. M. Evans, Gillespie, & Martin, 2002). The effect of *C* is to make children reared together similar on the trait of interest; both *C* and *A* contribute to sibling similarity. *E* represents elements of the environment that uniquely affect individuals and therefore contribute to differences between twins. Measurement error is included in the *E* term.

#### Genetic analyses

**Fig. 1.**
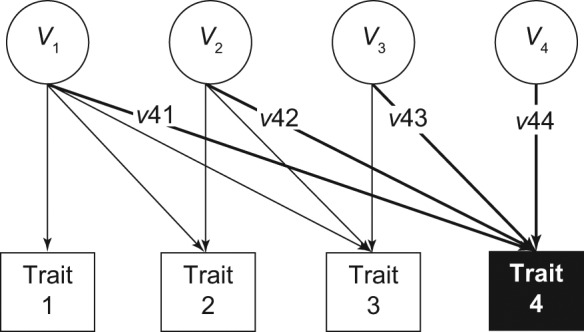
Schematic illustrating Cholesky decomposition. Measured traits (Traits 1–4) are regressed on corresponding latent variables (*V*_1_–*V*_4_). *V*_1_ is the total variation in Trait 1 and takes precedence in explaining variance in Traits 2, 3, and 4. *V*_2_ is the residual variance in Trait 2 and has next priority in explaining variance in Traits 3 and 4. *V*_3_ is the residual variance in Trait 3 and has next priority in explaining variance in Trait 4. *V*_4_ explains residual variance in Trait 4. Each measured trait is regressed on all preceding latent variables, and all latent variables are uncorrelated. The total variation in Trait 4 is estimated by squaring and summing the paths from *V*_1_ through *V*_4_ to Trait 4 (*v*41 through *v*44, respectively). *V*_1_ through *V*_4_ can be decomposed into additive genetic (*A*), shared environmental (*C*), and nonshared environmental (*E*) components. For example, the total additive genetic variance in Trait 4 would be explained by the squared and summed paths *a*41 through *a*44 from latent variables *A*_1_ through *A*_4_.

We used Cholesky decomposition models implemented in the OpenMx library (Boker et al., 2011) in the R statistical computing environment (R Development Core Team, 2011) to decompose the covariance structure of the relation between disruptive behaviors and CHAOS scores at ages 9 and 12. All available data were included in the models using full information maximum likelihood. Figure 1 shows a path diagram of the Cholesky decomposition used to model the cross-lagged effects of a pair of traits.

In a Cholesky decomposition, each subsequent observed variable is regressed on the latent *A, C*, and *E* variance components of all the previous observed variables. In Figure 1, *V*_1_ explains the total variance in Trait 1 (i.e., *A*_1_ + *C*_1_ + *E*_1_). Traits 2, 3, and 4 are regressed on the latent variable *V*_1_; in other words, the variance component *V*_1_ takes precedence in explaining variance in these three measured traits. *V*_2_ then explains the residual variance in Trait 2 (*A*_2_ + *C*_2_ + *E*_2_), that is, variance not correlated with *V*_1_. *V*_2_ also has next priority in explaining variance in Trait 3 and Trait 4. *V*_3_ and *V*_4_ explain residual variance in Traits 3 and 4, respectively, and are uncorrelated with each other or with *V*_2_ and *V*_1_. The total genetic variation in Trait 4 is estimated by squaring and summing the genetic paths (*a*41–*a*44) from the *A* components of *V* (*A*_1_–*A*_4_) to Trait 4. Similarly, shared and unique environmental variation in Trait 4 is estimated by squaring and summing the paths from all *C* and *E* components, respectively.

To determine whether genetic influences on disruptive behaviors at age 9 accounted for genetic influences on CHAOS at age 12 (controlling for CHAOS at age 9), we ordered the traits in the Cholesky decomposition so that Trait 1 was CHAOS scores at age 9, Trait 2 was disruptive behavior at age 9, Trait 3 was disruptive behavior at age 12, and Trait 4 was CHAOS scores at age 12. The degree to which the genetic path between Trait 2 and Trait 4 accounted for the total genetic variation in CHAOS scores at age 12—*a*42^2^/(*a*41^2^ + *a*42^2^ + *a*43^2^ + *a*44^2^)—addressed our substantive research question.

Similarly, to determine whether environmental influences on CHAOS scores at age 9 accounted for environmental influences on disruptive behaviors at age 12 (controlling for disruptive behavior at age 9), we ordered the traits so that Trait 1 was disruptive behavior at age 9, Trait 2 was CHAOS scores at age 9, Trait 3 was CHAOS scores at age 12, and Trait 4 was disruptive behavior at age 12. The full bivariate cross-lagged model was achieved by running the Cholesky decompositions with these alternative trait orderings (Luo, Haworth, & Plomin, 2010).

## Results

### Descriptive statistics

Descriptive statistics and results from analyses of variance are presented in Table 1. Across all measures, at both ages, there was no indication of substantial sex or zygosity differences: Main and interactive effects of sex and zygosity accounted for 6% or less of the variance in CHAOS scores, conduct problems, or hyperactivity-inattention. Because similarity due to age and sex can contribute to phenotypic twin similarity and inflate estimates of *C*, the measures were corrected for the effects of age and sex, as is standard practice in the analysis of twin data (McGue & Bouchard, 1984).

**Table 1.** Means for the Key Variables and Results of Sex × Zygosity Analyses of Variance

							ANOVA results			
Score	*n*	Overall	Female twins	Male twins	Monozygotic twins	Dizygotic twins	Sex (*p*)	Zygosity (*p*)	Sex × Zygosity interaction (*p*)	*R*^2^
CHAOS at age 9	3,136	4.46 (2.32)	4.31 (2.26)	4.63 (2.37)	4.44 (2.33)	4.48 (2.31)	<.01	.79	.47	<.01
Conduct at age 9	3,264	1.26 (1.42)	1.10 (1.30)	1.44 (1.53)	1.27 (1.46)	1.25 (1.40)	<.01	.49	.05	.02
Hyperactivity at age 9	3,261	3.18 (2.34)	2.74 (2.08)	3.68 (2.51)	3.27 (2.29)	3.13 (2.37)	<.01	.03	.75	.04
CHAOS at age 12	5,501	4.01 (2.05)	3.91 (2.06)	4.12 (2.03)	4.01 (2.04)	4.01 (2.05)	<.01	.79	.07	<.01
Conduct at age 12	5,592	1.32 (1.45)	1.21 (1.38)	1.44 (1.51)	1.30 (1.42)	1.33 (1.46)	<.01	.50	.28	.01
Hyperactivity at age 12	5,591	2.81 (2.25)	2.30 (2.01)	3.38 (2.37)	2.82 (2.20)	2.80 (2.28)	<.01	.38	.70	.06

Note: Standard deviations are given in parentheses. The statistics in this table were calculated using data from one randomly selected member of each twin pair. CHAOS = Confusion, Hubbub, and Order Scale (Matheny, Wachs, Ludwig, & Phillips, 1995); ANOVA = analysis of variance.

Phenotypic correlations among CHAOS scores and disruptive behavior are shown in Table 2. Correlations within traits across time were moderate for CHAOS scores (*r* = .45) and high for disruptive behaviors (conduct: *r* = .56; hyperactivity-inattention: *r* = .66); correlations between CHAOS scores and disruptive behavior across trait and time were modest (*r*s = .20–.24).

**Table 2.** Phenotypic (Pearson’s) Correlations and Intraclass Twin Correlations

Type of correlation and variable	CHAOS scores at age 9	Conduct at age 9	Hyperactivity at age 9	CHAOS scores at age 12	Conduct at age 12	Hyperactivity at age 12
Phenotypic correlations^a^						
Conduct at age 9	.27 (3,109)	—	—	—	—	—
Hyperactivity at age 9	.25 (3,106)	.49 (3,261)	—	—	—	—
CHAOS scores at age 12	.45 (2,489)	.23 (2,587)	.20 (2,584)	—	—	—
Conduct at age 12	.21 (2,522)	.56 (2,625)	.35 (2,622)	.26 (5,448)	—	—
Hyperactivity at age 12	.24 (2,521)	.42 (2,624)	.66 (2,621)	.24 (5,447)	.47 (5,591)	—
Intraclass correlations by sex and zygosity						
Monozygotic (all)	.66 [.63, .69]	.80 [.78, .82]	.73 [.70, .76]	.65 [.62, .67]	.77 [.75, .79]	.75 [.73, .77]
Dizygotic (all)	.52 [.49, .56]	.49 [.46, .53]	.15 [.11, .19]	.56 [.54, .58]	.49 [.46, .51]	.27 [.23, .30]
Monozygotic male	.64 [.58, .69]	.81 [.78, .83]	.75 [.71, .78]	.61 [.56, .65]	.75 [.72, .78]	.75 [.72, .78]
Dizygotic male	.55 [.48, .61]	.52 [.45, .58]	.16 [.08, .25]	.57 [.52, .62]	.50 [.44, .55]	.24 [.18, .30]
Monozygotic female	.68 [.64, .72]	.79 [.76, .81]	.71 [.67, .75]	.68 [.64, .71]	.79 [.77, .81]	.76 [.73, .78]
Dizygotic female	.56 [.50, .62]	.57 [.52, .63]	.15 [.07, .23]	.61 [.57, .65]	.55 [.50, .59]	.30 [.25, .36]
Dizygotic same sex	.56 [.51, .60]	.55 [.51, .59]	.16 [.10, .22]	.60 [.56, .62]	.53 [.49, .56]	.28 [.24, .32]
Dizygotic opposite sex	.49 [.44, .54]	.44 [.39, .49]	.14 [.08, .20]	.52 [.49, .56]	.44 [.40, .48]	.25 [.21, .30]

Note: For phenotypic correlations, the number of observations is given in parentheses; for intraclass correlations, 95% confidence intervals are shown. Pearson’s correlations were calculated using one randomly selected member of each twin pair. CHAOS = Confusion, Hubbub, and Order Scale (Matheny, Wachs, Ludwig, & Phillips, 1995).

a All phenotypic correlations were significant (*p* < .001).

### Phenotypic evidence for cross-lagged effects

Standardized parameter estimates from a series of ordinary least squares regression analyses showed evidence of cross-lagged effects. Both age-9 measures were entered simultaneously. Both conduct problems (β = 0.07, *p* < .001) and hyperactivity-inattention (β = 0.08, *p* < .001) at age 12 were significantly predicted by CHAOS scores at age 9, even after controlling for the effects of conduct problems and hyperactivity-inattention at age 9. The reverse was also true: CHAOS scores at 12 were predicted by conduct problems (β = 0.12, *p* < .001) and hyperactivity (β = 0.09, *p* < .001) at age 9, even after controlling for the effects of CHAOS scores at age 9.

### Genetically sensitive analyses

We performed full sex-limitation univariate analyses on disruptive behavior and CHAOS scores at ages 9 and 12 to estimate the genetic and environmental variance components separately for males and females. Overall, the models estimating genetic and environmental parameters separately for males and females did not provide a significantly better fit to the data than the more parsimonious scalar model did. The latter estimated one value of *A, C*, and *E* for both males and females by accounting for sex differences in the phenotypic variance. Although sex-limitation modeling suggested a lower genetic (or shared environmental) correlation in opposite-sex pairs than in same-sex pairs for hyperactivity-inattention at age 9, the *ACE* estimates for males and females were similar and had overlapping confidence intervals. We explored this potential difference in the multivariate analyses described in the following section. *ACE* estimates derived from the univariate scalar models showed that genetic and unique environmental factors accounted for significant variance in all three measures at ages 9 and 12. Shared environmental factors accounted for significant variance in CHAOS scores and conduct problems at ages 9 and 12, but not in hyperactivity-inattention.

### Multivariate analyses of the links between CHAOS scores and disruptive behavior

Because of the limited evidence of sex differences in univariate estimates, all multivariate analyses were conducted for males and females combined (with the inclusion of a scalar to account for differences in phenotypic variance between boys and girls). However, we also applied the same multivariate analyses to males and females separately. Conclusions drawn from the separated-by-sex analyses are limited because comparisons are made between groups (sexes) with different phenotypic variances. Nonetheless, we have noted whether the multivariate results changed when analyzed separately by sex.^1^

**Fig. 2.**
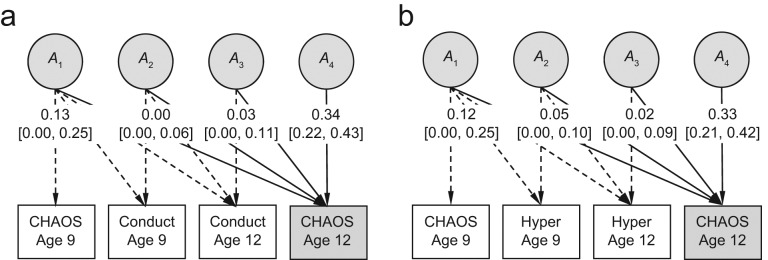
Cholesky decomposition showing the effects of genetic variance (*A*) in disruptive behavior on Confusion, Hubbub, and Order Scale (CHAOS; Matheny, Wachs, Ludwig, & Phillips, 1995) scores at age 12. Separate models are shown for the effects of (a) conduct and (b) hyperactivity-inattention (“Hyper”), with both analyses controlling for the genetic effects of CHAOS scores at age 9. *A*_1_ captures the total genetic variation in CHAOS scores at age 9; *A*_2_, *A*_3_, and *A*_4_ are the residual genetic variances in disruptive behavior at age 9, disruptive behavior at age 12, and CHAOS scores at age 12, respectively. Standardized (unsquared) path coefficients and 95% confidence intervals are shown.

**Fig. 3.**
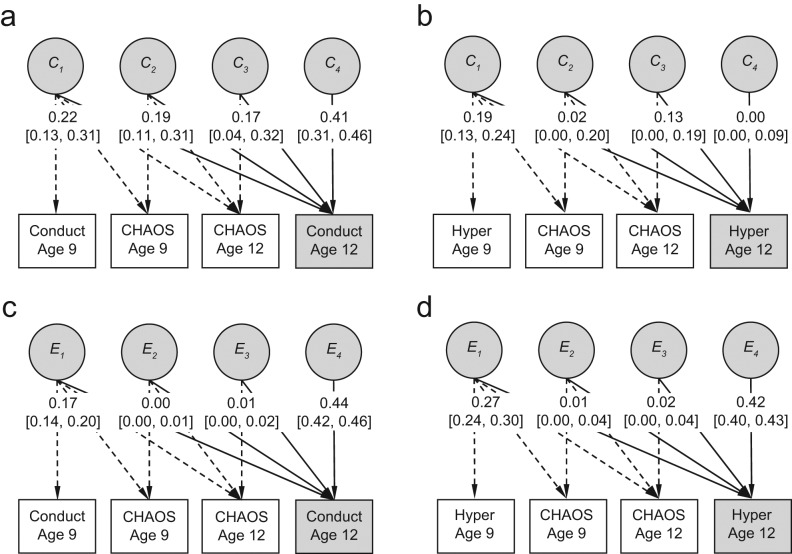
Cholesky decomposition showing the effects of shared environmental (*C*) and nonshared environmental (*E*) variance in Confusion, Hubbub, and Order Scale (CHAOS; Matheny, Wachs, Ludwig, & Phillips, 1995) scores on disruptive behavior at age 12. Separate models are shown for the effects on conduct at age 12 (a, c) and on hyperactivity-inattention (“Hyper”) at age 12 (b, d). All analyses controlled for the corresponding disruptive behavior at age 9. *C*_1_ and *E*_1_ refer to the total environmental variance in disruptive behavior at age 9; *C*_2_, *C*_3_, and *C*_4_ and *E*_2_, *E*_3_, and *E*_4_ refer to the residual environmental variance in CHAOS scores at age 9, CHAOS scores at age 12, and disruptive behavior at age 12, respectively. Standardized (unsquared) path coefficients and 95% confidence intervals are shown.

The salient results from our multivariate modeling of the cross-lagged relation between CHAOS scores and disruptive behavior are presented in this article. Standardized (unsquared) partial regression coefficients show the effect of latent genetic components of disruptive behavior on CHAOS scores (Fig. 2) and latent environmental effects of CHAOS scores on disruptive behavior (Fig. 3). Tables S1 and S2 in the Supplemental Material available online include point estimates and 95% confidence intervals for all path estimates.

### What explains genetic influences on CHAOS scores at age 12?

The total genetic variation in CHAOS scores at age 12 was derived by squaring and summing the path coefficients that led from the genetic factors (*A*_1_ through *A*_4_) to CHAOS scores at age 12 (Fig. 2). In the relation between conduct problems and CHAOS scores, the total genetic variation on CHAOS scores at age 12 is given by the formula 0.13^2^ + 0.00^2^ + 0.03^2^ + 0.34^2^ = 0.1334, or 13%. Of this total genetic variation, about 13% was carried over from genetic influences on CHAOS scores at age 9 (0.13^2^/0.1334), and 87% was specific to CHAOS scores at age 12 years (0.34^2^/0.1334). Less than 1% was explained by genetic influences on conduct problems at ages 9 and 12; these paths (from *A*_2_ and *A*_3_) were not statistically significant (i.e., 95% confidence intervals included 0). Similar to the results for conduct problems, results for genetic influences on hyperactivity-inattention at ages 9 and 12 explained a nonsignificant 2% of the genetic variation in CHAOS scores at age 12. When separated by sex, results were similar for both males and females and comparable with the combined analyses.

### What explains environmental influences on disruptive behavior problems?

Figure 3 shows the shared (*C*) and nonshared (*E*) environmental influences of CHAOS scores on disruptive behavior at age 12, after accounting for the effects of disruptive behavior at age 9. Shared environmental factors accounted for 28% of the variation in conduct problems at age 12. Shared environmental influences on CHAOS scores at ages 9 and 12 explained 13% and 10% of this total variation, respectively. Results of the univariate scalar model showed that shared environmental influences on hyperactivity-inattention at age 12 were statistically nonsignificant. However, multivariate analyses—which benefitted from the additional information of covariances between traits—suggested a small shared environmental component in hyperactivity-inattention at age 12 (about 5%), whose only significant contribution was from shared environmental influences on hyperactivity-inattention at age 9.

Although unique environmental factors accounted for 22% of the variation in conduct problems at age 12 and 25% of the variation in hyperactivity-inattention, virtually none of this variation was explained by unique environmental influences on CHAOS scores at ages 9 or 12.

Sex-specific multivariate analyses suggested a nonsignificant difference between boys and girls in the shared environmental link between CHAOS scores at age 9 and conduct problems at age 12. However, given that neither of these variables showed univariate sex differences, separating the sample by sex may have simply reduced power to detect environmental effects.

## Discussion

Consistent with previous results using the TEDS sample, our analyses identified genetic and environmental influences on measures of household chaos, conduct problems, and hyperactivity-inattention at ages 9 and 12. The goal of our analyses was to identify the developmental origins of those genetic and environmental influences. Specifically, we tested whether genetic influences on disruptive behaviors at age 9 explained genetic variation in CHAOS scores at age 12 and whether environmental influences on CHAOS scores at age 9 explained environmental variation in disruptive behaviors at age 12.

We found that shared environmental influences on CHAOS scores at age 9 uniquely accounted for 13% of the shared environmental variation in conduct problems at age 12, which suggests that some of the cross-lagged effect of CHAOS scores on subsequent conduct problems was environmentally mediated. This finding suggests that encouraging parents to adopt stable routines and to minimize extraneous noise in the house could complement other techniques used in parent-training programs to prevent children’s disruptive behaviors, such as reinforcing children’s prosocial behaviors and reducing the use of harsh, coercive discipline.

Although genetic influences on disruptive behaviors were substantial, they accounted for little of the genetic variation in CHAOS scores at age 12. Although other researchers have identified similarly small contributions of disruptive behaviors to the heritability of the family environment (Burt et al., 2005; Larsson et al., 2008), our findings could be due to how CHAOS scores were measured. The fact that CHAOS scores were measured for each twin in a pair generates two possibilities for what it means for CHAOS scores to be heritable. One possibility is that genetic influences on CHAOS scores reflect genetically based individual differences among children (e.g., disruptive behavior problems) that elicit a chaotic environment. A second possibility is that genetic influences on CHAOS scores reflect genetically based differences in children’s perceptions of the environment. If the latter possibility is correct, then the degree to which children differ in their reports of household chaos may have more to do with how attentive or sensitive they are to their surroundings—characteristics that are not necessarily captured by children’s disruptive behaviors as well as they might be by a measure of stress reactivity, for example. In reality, genetic influences on CHAOS scores are likely to reflect both genetically based differences in children’s behaviors as well as their perceptions.

Although the genetic cross-lagged analysis provides a direct estimate of the genetic and environmental influences on the cross-lagged paths—which was our goal—it does not allow for the simultaneous estimation of both cross-lagged paths in the same model (Luo et al., 2010). In contrast, the model developed by Burt et al. (2005) estimates a fully cross-lagged model of the relation among the phenotypes. Although the cross-lagged model reported by Burt et al. (2005) has the advantage of being economical (in that it models the bidirectional relation in a single run), it does not directly decompose the stability (across time, within trait) and cross-lagged (across time, across trait) effects into *ACE* components. Estimates of the *ACE* effects transmitted along stable and cross-lagged paths are simply scalar multiples of the *ACE* effects at the earlier time point, constrained to be in the *ACE* proportions at the earlier time point (Luo et al., 2010). Because we required direct estimates of genetic and environmental influences on the cross-lagged paths to answer our focal research questions, we opted to use the Cholesky approach. A second limitation of our study was that the internal consistency of the CHAOS measures was only moderate. However, parent and child reports of CHAOS were highly correlated, which provided additional validity for the measure.

In conclusion, although individual differences in reports of environmental confusion were partly genetic in origin, this genetic variance was not accounted for by the heritable component of children’s disruptive behavior. In addition, the effects of environmental confusion on children’s disruptive behaviors were environmentally mediated. Noisy, crowded homes characterized by a lack of routines may undermine children’s ability to regulate emotions and behavior and may provide children with opportunities to act out.

## Supplementary Material

Supplementary Material

## Notes

1. Full details of the sex-specific analyses, as well as the univariate sex-limitation modeling, are available from the corresponding author.

## References

[bibr1-0956797611431693] Boker S. Neale M. Maes H. Wilde M. Spiegel M. Brick T.. . . Fox J. (2011). OpenMx: An open source extended structural equation modeling framework. Psychometrika, 76, 306–317 10.1007/s11336-010-9200-6 PMC3525063 23258944

[bibr2-0956797611431693] Burt S. A. McGue M. Krueger R. F. Iacono W. G. (2005). How are parent-child conflict and child externalizing symptoms related over time? Results from a genetically informative cross-lagged study. Development and Psychopathology, 17, 145–165 15971764 10.1017/S095457940505008X PMC2245887

[bibr3-0956797611431693] Deater-Deckard K. Mullineaux P. Y. Beekman C. Petrill S. A. Schatschneider C. Thompson L. A. (2009). Conduct problems, IQ, and household chaos: A longitudinal multi-informant study. Journal of Child Psychology and Psychiatry, 50, 1301–1308 19527431 10.1111/j.1469-7610.2009.02108.x PMC3217298

[bibr4-0956797611431693] Dumas J. E. Nissley J. Nordstrom A. Smith E. P. Prinz R. J. Levine D. W. (2005). Home chaos: Sociodemographic, parenting, interactional, and child correlates. Journal of Clinical Child and Adolescent Psychology, 34, 93–104 15677284 10.1207/s15374424jccp3401_9

[bibr5-0956797611431693] Duncan G. J. Brooks-Gunn J. (1997). Consequences of growing up poor. New York, NY: Russell Sage

[bibr6-0956797611431693] Evans D. M. Gillespie N. A. Martin N. G. (2002). Biometrical genetics. Biological Psychology, 61, 33–51 12385668 10.1016/s0301-0511(02)00051-0

[bibr7-0956797611431693] Evans G. W. (2006). Child development and the physical environment. Annual Review of Psychology, 57, 423–451 10.1146/annurev.psych.57.102904.190057 16318602

[bibr8-0956797611431693] Evans G. W. Gonnella C. Marcynyszyn L. A. Gentile L. Salpekar N. (2005). The role of chaos in poverty and children’s socioemotional adjustment. Psychological Science, 16, 560–565 16008790 10.1111/j.0956-7976.2005.01575.x

[bibr9-0956797611431693] Evans G. W. Maxwell L. E. Hart B. (1999). Parental language and verbal responsiveness to children in crowded homes. Developmental Psychology, 35, 1020–1023 10442870 10.1037//0012-1649.35.4.1020

[bibr10-0956797611431693] Goodman R. (1997). The Strengths and Difficulties Questionnaire: A research note. Journal of Child Psychology and Psychiatry, 38, 581–586 9255702 10.1111/j.1469-7610.1997.tb01545.x

[bibr11-0956797611431693] Hanscombe K. B. Haworth C. M. A. Davis O. S. P. Jaffee S. R. Plomin R. (2010). The nature (and nurture) of children’s perceptions of family chaos. Learning and Individual Differences, 20, 549–553 21572559 10.1016/j.lindif.2010.06.005 PMC3091813

[bibr12-0956797611431693] Hanscombe K. B. Haworth C. M. A. Davis O. S. P. Jaffee S. R. Plomin R. (2011). Chaotic homes and school achievement: A twin study. Journal of Child Psychology and Psychiatry, 52, 1212–1220 21675992 10.1111/j.1469-7610.2011.02421.x PMC3175268

[bibr13-0956797611431693] Larsson H. Viding E. Rijsdijk F. V. Plomin R. (2008). Relationships between parental negativity and childhood antisocial behavior over time: A bidirectional effects model in a longitudinal genetically informative design. Journal of Abnormal Child Psychology, 36, 633–645 17602294 10.1007/s10802-007-9151-2

[bibr14-0956797611431693] Luo Y. L. L. Haworth C. M. A. Plomin R. (2010). A novel approach to genetic and environmental analysis of cross-lagged associations over time: The cross-lagged relationship between self-perceived abilities and school achievement is mediated by genes as well as the environment. Twin Research and Human Genetics, 13, 426–436 20874463 10.1375/twin.13.5.426 PMC3819564

[bibr15-0956797611431693] Matheny A. P. Wachs T. D. Ludwig J. Phillips K. (1995). Bringing order out of chaos: Psychometric characteristics of the Confusion, Hubbub, and Order Scale. Journal of Applied Developmental Psychology, 16, 429–444

[bibr16-0956797611431693] McGue M. Bouchard T. J. (1984). Adjustment of twin data for the effects of age and sex. Behavior Genetics, 14, 325–343 6542356 10.1007/BF01080045

[bibr17-0956797611431693] Muris P. Meesters C. van den Berg F. (2003). The Strengths and Difficulties Questionnaire (SDQ): Further evidence for its reliability and validity in a community sample of Dutch children and adolescents. European Child and Adolescent Psychiatry, 12, 1–8 12601558 10.1007/s00787-003-0298-2

[bibr18-0956797611431693] Oliver B. R. Plomin R. (2007). Twins’ Early Development Study (TEDS): A multivariate, longitudinal genetic investigation of language, cognition and behavior problems from childhood through adolescence. Twin Research and Human Genetics, 10, 96–105 17539369 10.1375/twin.10.1.96

[bibr19-0956797611431693] Plomin R. DeFries J. C. McClearn G. E. McGuffin P. (2008). Behavioral genetics (5th ed.). New York, NY: Worth Publishers

[bibr20-0956797611431693] Price T. S. Freeman B. Craig I. Petrill S. A. Ebersole L. Plomin R. (2000). Infant zygosity can be assigned by parental report questionnaire data. Twin Research, 3, 129–133 11035484 10.1375/136905200320565391

[bibr21-0956797611431693] R Development Core Team (2011). R: A language and environment for statistical computing [Computer software]. Available from www.r-project.org/

[bibr22-0956797611431693] Rijsdijk F. V. Sham P. (2002). Analytic approaches to twin data using structural equation models. Briefings in Bioinformatics, 3, 119–133 12139432 10.1093/bib/3.2.119

[bibr23-0956797611431693] Wachs T. D. (1989). The nature of the physical microenvironment: An expanded classification system. Merrill-Palmer Quarterly: Journal of Developmental Psychology, 35, 399–419

[bibr24-0956797611431693] Wohlwill J. Heft H. (1987). The physical environment and development of the child. In Stokol D. Altman I. (Eds.), Handbook of environmental psychology (pp. 281–328). New York, NY: Plenum Press

