# Supplementary material for: Chaotic Homes and Children’s Disruptive Behavior: A Longitudinal Cross-Lagged Twin Study
Source: Psychol Sci. 2012 Jun;23(6):643–50. doi: 10.1177/0956797611431693 (PMC3494454; doi:10.1177/0956797611431693)
Supplement: Supplementary Material [file DS_10.1177_0956797611431693.pdf]

Table 1s. Cholesky decomposition of the cross-lagged relationship between **parent-rated conduct and child-rated CHAOS** (standardized un-squared path estimates)

Variance Component	*9-year*		*12-year*	
	**CHAOS**	**conduct**	**conduct**	**CHAOS**
**A_1**	.49 (.39-.56)	.14 (.03-.25)	.07 (.00-.18)	.13 (.00-.25)
**C_1**	.65 (.59-.70)	.31 (.24-.39)	.30 (.22-.38)	.59 (.51-.67)
**E_1**	.58 (.56-.61)	.00 (.00-.03)	.00 (.00-.02)	.02 (.00-.06)
**A_2**		.71 (.66-.76)	.54 (.47-.59)	.00 (.00-.06)
**C_2**		.41 (.32-.49)	.05 (.00-.17)	.10 (.00-.20)
**E_2**		.45 (.43-.47)	.17 (.14-.20)	.00 (.00-.02)
**A_3**			.46 (.38-.52)	.03 (.00-.11)
**C_3**			.43 (.34-.48)	.13 (.00-.24)
**E_3**			.44 (.42-.46)	.01 (.00-.03)
**A_4**				.34 (.22-.43)
**C_4**				.37 (.19-.47)
**E_4**				.60 (.58-.62)

	**conduct**	**CHAOS**	**CHAOS**	**conduct**
**A_1**	.73 (.68-.78)	.09 (.03-.16)	.02 (.00-.08)	.54 (.47-.60)
**C_1**	.52 (.45-.58)	.39 (.30-.50)	.45 (.35-.55)	.22 (.13-.31)
**E_1**	.45 (.43-.47)	.00 (.00-.04)	.00 (.00-.02)	.17 (.14-.20)
**A_2**		.48 (.38-.56)	.13 (.00-.27)	.00 (.00-.07)
**C_2**		.52 (.40-.60)	.39 (.24-.52)	.19 (.11-.31)
**E_2**		.58 (.56-.61)	.02 (.00-.06)	.00 (.00-.01)
**A_3**			.34 (.21-.43)	.04 (.00-.17)
**C_3**			.39 (.22-.49)	.17 (.04-.32)
**E_3**			.60 (.58-.62)	.01 (.00-.02)
**A_4**				.46 (.38-.52)
**C_4**				.41 (.31-.46)
**E_4**				.44 (.42-.46)

In each row are the standardized (unsquared) path coefficients (and 95% confidence interval) leading from the latent variance component in column 1 to the measured trait labeled at the top of each column. Variance components with subscript 1 explain variance in measured trait 1 (column 2); subscripts 2, 3, and 4 denote variance components explaining residual variation in measured traits 2, 3, and 4 (columns 3, 4, and 5). A, C, and E = genetic, shared, and nonshared environmental variance components**;** CHAOS = CHAOS; conduct = conduct problems.

Table 2s. Cholesky decomposition of the cross-lagged relationship between **parent-rated hyperactivity and child-rated CHAOS** (standardized un-squared estimates)

Component	***9-year***		***12-year***	
	**CHAOS**	**hyperactivity**	**hyperactivity**	**CHAOS**
**A_1**	.47 (.38-.56)	.20 (.12-.29)	.23 (.14-.34)	.12 (.00-.25)
**C_1**	.66 (.60-.71)	.21 (.16-.26)	.19 (.13-.24)	.58 (.50-.67)
**E_1**	.59 (.56-.61)	.00 (.00-.01)	.01 (.00-.04)	.02 (.00-.06)
**A_2**		.79 (.76-.81)	.53 (.49-.57)	.05 (.00-.10)
**C_2**		.02 (.00-.11)	.13 (.00-.20)	.41 (.00-.51)
**E_2**		.54 (.51-.57)	.27 (.24-.30)	.00 (.00-.01)
**A_3**			.60 (.56-.63)	.02 (.00-.09)
**C_3**			.00 (.00-.19)	.00 (.00-.50)
**E_3**			.41 (.40-.43)	.03 (.00-.06)
**A_4**				.33 (.21-.42)
**C_4**				.00 (.00-.26)
**E_4**				.60 (.58-.62)

	**hyperactivity**	**CHAOS**	**CHAOS**	**hyperactivity**
**A_1**	.82 (.79-.84)	.12 (.07-.16)	.09 (.04-.13)	.58 (.54-.61)
**C_1**	.22 (.17-.26)	.65 (.57-.70)	.59 (.46-.68)	.19 (.13-.24)

**E_1**	.54 (.51-.57)	.00 (.00-.01)	.00 (.00-.01)	.27 (.24-.30)
**A_2**		.46 (.37-.54)	.10 (.00-.21)	.09 (.00-.19)
**C_2**		.00 (.00-.32)	.05 (.00-.55)	.02 (.00-.20)
**E_2**		.59 (.56-.61)	.02 (.00-.06)	.01 (.00-.04)
**A_3**			.34 (.22-.42)	.04 (.00-.15)
**C_3**			.40 (.21-.50)	.13 (.00-.19)
**E_3**			.60 (.58-.62)	.02 (.00-.04)
**A_4**				.60 (.56-.63)
**C_4**				.00 (.00-.09)
**E_4**				.42 (.40-.43)

See note to Table 1s; hyperactivity = hyperactivity/inattention.
